# The Higher Plasma Malondialdehyde Concentrations Are Determined by Metabolic Syndrome-Related Glucolipotoxicity

**DOI:** 10.1155/2014/505368

**Published:** 2014-06-24

**Authors:** Fernando Moreto, Erick P. de Oliveira, Rodrigo M. Manda, Roberto C. Burini

**Affiliations:** ^1^Center for Nutritional and Exercise Metabolism (CeMENutri), Botucatu School of Medicine, Sao Paulo State University, Botucatu, SP, Brazil; ^2^School of Medicine, Federal University of Uberlandia, Uberlandia, MG, Brazil

## Abstract

This study aimed to elucidate the determinants of higher plasma malondialdehyde (MDA) in free-living adults. In a cross-sectional study we evaluated 148 free-living subjects (54 ± 11 years, 78% women) at high risk for or with metabolic syndrome (MetS). They were assessed by anthropometry and body composition, dietary intake, and clinical and laboratorial analysis. The analysis of plasma MDA was performed by HPLC, and concentration values were used to provide four groups according to percentile distribution. Subjects with higher plasma MDA showed higher prevalence of MetS and higher values of waist circumference (WC), glucose, triglycerides (TG), *γ*-glutamyltransferase (*γ*-GT), and higher energy intake. Multiadjusted logistic regression analysis identified as determinants of higher plasma MDA the altered values of WC and *γ*-GT followed by hypertriglyceridemia, hyperglycemia, insulin resistance, higher dietary sugar-intake, and presence of MetS. In conclusion, the glucolipotoxic state predisposed by the presence of MetS seems to be the major determinant of higher plasma MDA concentrations.

## 1. Introduction

Lipid peroxidation (LPO) is a phenomenon where instable molecules are responsible for oxidizing lipids, proteins, and nucleic acids, resulting in cell malfunctions with generalized responses [1]. LPO has been characterized as a natural process of lipid degradation. In cell membranes, LPO begins when electrons from lipids are kidnapped by unstable free radicals promoting a chain reaction with successive oxidations that results in lipid instability and formation of by-products such as malondialdehyde (MDA) [2]. MDA is formed by enzymatic and/or free-radical peroxidation of PUFAs like arachidonic acid and docosahexaenoic acid by cleavage of its double bounds and releasing bis-aldehyde malonaldehyde [3]. The presence of factors accelerating free-radical production and loss or failure in neutralizing damaging processes (antioxidants) characterizes oxidative stress.

Several factors are associated with oxidative imbalance in the human organism. Among them, behavioral (e.g., smoking, nutrition, and exercise) and pathological (e.g., metabolic syndrome, type 2 diabetes, and dyslipidemia) factors can be pointed out. Epidemiological evidences have shown associations between dietary sugar-intake and increased risk for developing metabolic syndrome [4], type 2 diabetes [5], obesity [5, 6], and body adiposity [7], and the pathophysiology of these complications includes ectopic fat deposition with glucotoxic and lipotoxic actions [8]. To our knowledge there are few studies evaluating direct effects on LPO accessed by plasma MDA concentrations in humans. Plasma MDA can be easily assessed in large-scale people groups. Thus, knowledge of the interaction between behavioral and pathological processes in the initiation of lipoperoxidative activity can generate important tools for the prevention of pathological processes derived from lifestyle. So, this study aimed to elucidate the determinants of the higher plasma MDA concentrations in free-living adults at high risk for or with MetS.

## 2. Methods

### 2.1. Study Design and Subjects

The subjects were beginners at the Botucatu Longitudinal Study (BLS) on Healthy Lifestyle Promotion Program called “Move for Health” as primary care for chronic noncommunicable diseases. This program is conducted by multidisciplinary staff from Center for Nutritional and Exercise Metabolism (CeMENutri) at UNESP Medical School (Botucatu, SP, Brazil). In this cross-sectional study with convenience sample, 541 adults were admitted to the program and 278 subjects were eligible for the study. The inclusion criteria were 35 years old or older and at high risk for (or presenting) MetS, without history of complications from cardiovascular, hepatic, renal, inflammatory, and autoimmune diseases or cerebral stroke. We excluded subjects who did not complete all assessments and those using vitamin supplements, inflammatory drugs, and chronic alcoholics. One hundred and thirty subjects did not achieve inclusion criteria or were excluded, so 148 subjects were included in this study. Written informed consent was obtained from all subjects and this study was conducted according to the guidelines laid down in the Declaration of Helsinki. All procedures involving human subjects were approved by the Ethics Committee of the Botucatu School of Medicine (Of 557/2011).

### 2.2. Assessments

During medical evaluation subjects were screened for chronic diseases and submitted to assessments of physical activity readiness (PAR-Q). The presences of diseases or clinical history that would preclude the subjects' participation in the study according to inclusion and exclusion criteria were also taken. Also, at this time smoking status was self-reported by the subjects and the measures of systolic and diastolic blood pressures were made using the auscultatory method. Cardiorespiratory fitness was assessed estimating maximal oxygen consumption (VO_2max⁡_) by an equation proposed by the American College of Sports Medicine. This equation considers the total time of maximal treadmill test using the Balke protocol [9].

Body weight and height were obtained for subsequent Body Mass Index (BMI) calculation. Waist circumference (WC) was measured using a millimeter metal tape according to WHO recommendations. Body fat percentages and lean mass were estimated by equations considering electric resistance and reactance of the body provided by a bioelectric impedance device (Biodynamics, model 450, USA). Muscle mass in kilograms was estimated using the equation proposed by Janssen et al. [10], and these values were used to calculate the Muscle Mass Index in kg/m^2^.

Subjects were submitted to nutritional history through 24-hour recall. Dietary data obtained in household measures were converted to grams and milliliters to enable chemical analysis of food consumption. Subsequently, data were processed in a nutritional analysis program (NutWin, Support Program for Nutrition, version 1.5, UNIFESP, 2002). To assess the dietary quality we used the adapted Healthy Eating Index (HEI), compiled from the American HEI [11]. This index assesses the quality of the diet by assigning points according to the individual food intake [12].

Blood samples were obtained after overnight fasting by vacuum venipuncture. Laboratory analysis of lipid parameters (total and HDL-cholesterol and triglycerides), glucose, uric acid, and *γ*-glutamyltransferase (*γ*-GT) was performed within 4 hours after blood collection by dry chemistry method (Vitros 5600, Ortho Clinical Diagnostics, Johnson & Johnson Company, Raritan, NJ, USA). The LDL-cholesterol concentrations were estimated using the formula proposed by Friedewald. Serum concentrations of insulin were quantified by a chemiluminescent method (Immulite 2000, Siemens Healthcare Diagnostics, Marburg, Germany) and used for subsequent calculation of the Insulin Resistance Index HOMA-IR. Serum C-reactive protein (CRP) concentrations were measured by a high-sensitivity immunonephelometric assay (Siemens Healthcare Diagnostics, Marburg, Germany). Plasma MDA concentrations were performed by high performance liquid chromatography with fluorometric detection (HPLC, system LC10A, Shimadzu, Japan) as previously described [13].

The criteria used for MetS diagnosis were described by the American Heart Association/National Heart, Lung, and Blood Institute Scientific Statement [14].

### 2.3. Statistical Analysis

The statistical analysis was conducted with the statistical analysis software (SAS version 9.1.3, SAS Institute, USA), and the *α* of significance was set at 5% (*P* < 0.05). Initially, the normality of the data using the Kolmogorov-Smirnov test was tested. Data are presented as mean ± standard deviation (parametric variables) or median and interquartile range (nonparametric variables). The percentile values p25 (0.593 *μ*mol/L), p50 (0.857 *μ*mol/L), and p75 (1.058 *μ*mol/L) of plasma MDA were used to obtain four groups: very low MDA (those with plasma MDA < 0.593 *μ*mol/L), low MDA (those with plasma MDA between 0.593 and 0.856 *μ*mol/L), increased MDA (plasma MDA between 0.857 and 1.057 *μ*mol/L), and higher MDA (plasma MDA > 1.057 *μ*mol/L). The comparison among groups was performed by one-way ANOVA for parametric variables and Kruskal-Wallis ANOVA for nonparametric variables, both with Dunn's post hoc test when significant. The predictors for elevating plasma MDA in the presence of alterations were performed by multiadjusted logistic regression models (odds ratio (OR) with 95% confidence interval (CI)). For this analysis, all parameters were categorized and alterations were set as reference values according to age and gender. Adjustment models were also performed including age, gender, smoking status, medicine use, energy intake, and BMI.

## 3. Results

The sample was predominantly characterized by females (78%), aged 35 to 65 years old (70%), nonsmokers (76%), and overweight or obese (80%). The prevalence of MetS in this sample was 34%. Some subjects showed dyslipidemia (39%), hypertension (31%), and hyperglycemia (25%) and were under drug therapy (46%), which was considered when diagnosing MetS.

The higher plasma MDA group differed from other groups by showing higher values of WC, fasting blood glucose, TG, *γ*-GT, and energy intake. Total body fat, BMI, blood pressures, cardiorespiratory fitness, and cholesterol fractions were similar among groups (Table 1). Also, higher plasma MDA group was discriminated by showing higher and significant prevalence of MetS (50%) compared to other groups (29% in very low, 29% in low, and 38% in increased groups). Moreover, plasma MDA concentrations were significantly (*P* < 0.01) different between the presence (0.947 ± 0.339 *μ*mol/L) and absence (0.803 ± 0.283 *μ*mol/L) of MetS. Also, those with MetS showed higher dietary sugar-intake (0.83 [0.0–2.47] versus 0.5 [0.0–1.5], *P* < 0.01), higher WC measures (104.0 [93.5–110.9] versus 93.0 [85.0–104.0], *P* < 0.001), higher HOMA-IR (3.07 [1.63–5.77] versus 1.34 [0.89–2.32], *P* < 0.001) and higher TG (199.0 [148.0–248.5] versus 116.0 [88.3–139.8], *P* < 0.001), blood glucose (98.5 [90.0–125.0] versus 88.0 [82.0–94.8], *P* < 0.001), and *γ*-GT (33.0 [21.0–46.0] versus 23.0 [16.0–40.0], *P* < 0.01) concentrations than those without MetS.

**Figure 1:**
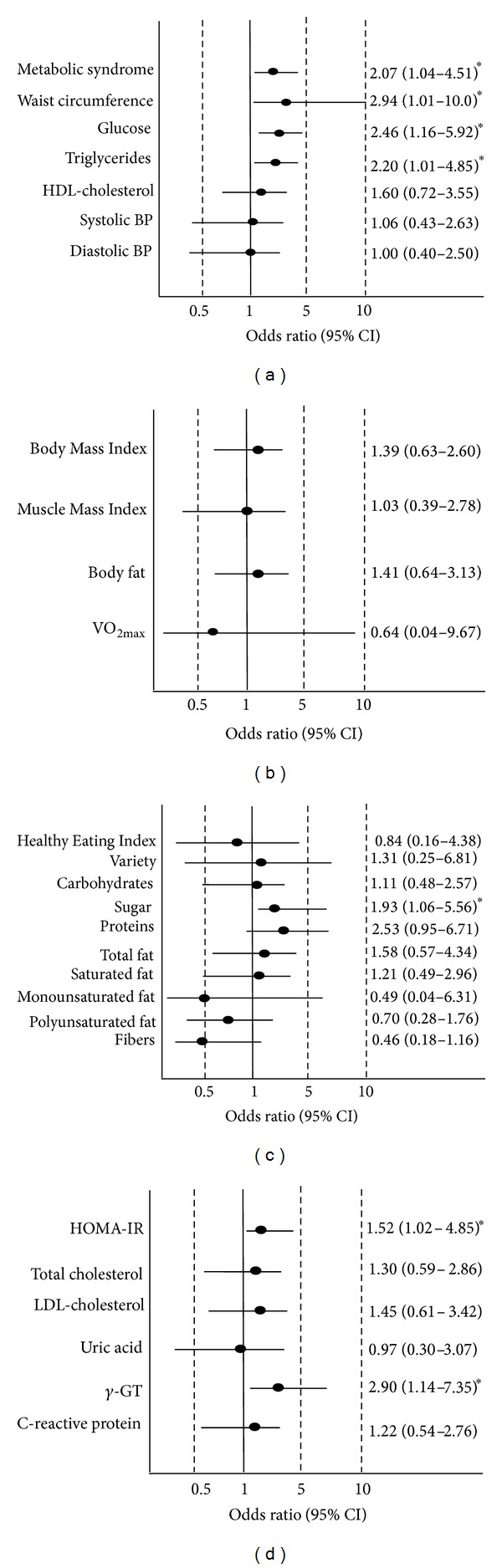
Logistic regression analysis identifying the main predictors for higher plasma MDA concentrations; (a) metabolic syndrome and its components; (b) anthropometry, body composition, and fitness; (c) dietary intake; (d) blood markers. Analyses were adjusted for age, gender, smoking status, medicine use, BMI, and energy intake. Black circles are odds ratio and traces are confidence interval. *P < 0.05.

The multiadjusted logistic regression analysis showed that MetS presence was identified as an independent predictor for higher plasma MDA concentrations (OR 2.07, CI 1.04 to 4.51). Likewise, alterations in MetS components such as WC (OR 2.94, CI 1.01 to 10.0), glucose (OR 2.46, CI 1.16 to 5.92), and TG (OR 2.20, CI 1.01 to 4.85) were also identified as predictors for higher plasma MDA (Figure 1(a)). BMI, muscle mass, and body fat showed no association with higher plasma MDA (Figure 1(b)); however, the higher values of HOMA-IR (OR 1.52, CI 1.02 to 4.85), *γ*-GT (OR 2.90, CI 1.14 to 7.35) (Figure 1(c)), and dietary sugar-intake (OR 1.93, CI 1.06 to 5.65) (Figure 1(d)) were also identified as predictors for higher plasma MDA concentrations.

## 4. Discussion

This study elucidated the major determinants of the higher plasma MDA concentrations in free-living adults at high risk for or with MetS. Altered values of WC and *γ*-GT were strongly associated with higher plasma MDA concentrations. Altered concentrations of TG and glucose, higher sugar/energy intake, insulin resistance, and the presence of MetS were also associated with higher plasma MDA concentrations. From the used plasma markers, blood glucose (and HOMA-IR), LDL-cholesterol, and TG are referred to as risk factors for lipoperoxidative activity with higher CRP (systemic inflammatory marker) and *γ*-GT (steatohepatitis marker) concentrations as its probably causes. On the other hand, higher plasma concentrations of uric acid are indicative of enhanced extracellular hydrosoluble antioxidant response whereas HDL-cholesterol presents both antioxidant and anti-inflammatory actions. From this point of view, these markers can be markedly influenced by lifestyle conditions like sedentary and inadequate nutrition.

Oxidative stress and chronic low-grade inflammation are common comorbidities of MetS. Age and gender showed no differences among plasma MDA groups whereas MetS prevalence was greater in subjects with higher plasma MDA concentrations. Increasing adiposity is determinant to the development of MetS with proinflammatory effects [15]. Hypertrophic adipocytes secrete cytokines (IL-6, TNF-*α*) and monocyte chemoattractants (MCP-1) and are characterized by macrophage infiltration generating global proinflammatory profile [16]. Additionally, macrophage activation leads to NADPH oxidase overexpression and activation, implicated in ROS production [17]. These ROS can oxidize the cell membrane lipids breaking their molecules with consequent increase in their plasma by-products. This proinflammatory state would be in conjunction with the occurrence of oxidative stress [18]; however, no associations between C-reactive protein concentrations and plasma MDA among groups were observed.

This study showed an independent association between higher dietary sugar-intake and higher plasma MDA, suggesting that sugar-intake is directly involved in the generation of oxidative stress. High sugar-intake induces hyperglycemic peaks with subsequent hyperinsulinemia [19]. We observed that hyperglycemia and HOMA-IR were associated with higher plasma MDA concentrations even after adjusting for smoking and obesity. Hyperglycemia-induced oxidative stress is characterized by the presence of advanced glycation end-products (AGEs) [20]. AGEs can oxidize lipids in cell membranes leading them to instability and consequent degradation to LPO by-products [21]. Besides MDA is considered a limited marker to assess overall oxidative stress; the analysis of plasma MDA performed by HPLC with fluorometric detection is very sensitive and widely used in scientific research assessing LPO [22]. Therefore, exposure to hyperglycemia and insulin resistance may be decisive for the development of LPO.

In the present study, subjects with higher dietary sugar-intake in our sample were characterized by increased intake of sweetened beverages including soft drinks (like soda) or industrialized fruit juices and candies. In Brazil, the predominant sugar-sweetening of these products is sucrose. An elegant meta-analysis showed that higher consumption of sweetened beverages is closely related to higher risk for developing MetS and type 2 diabetes [4]. Elevated sugar/energy intake is a predisposing condition to MetS development due to increasing adiposity [23] and the link between high sugar/energy intake and metabolic abnormalities seems to be the ectopic fat deposition [24]. Although cardiorespiratory fitness was not associated with plasma MDA concentrations, combating sedentary lifestyle with physical activity and nutritional adequacy can prevent fat deposition and MetS development, with consequent impact over plasma markers of oxidative stress [25].

The hypertriglyceridemic waist phenotype is considered a more simple way to diagnosing metabolic complications and is closely related to the development of insulin resistance and liver steatosis [26]. We observed an independent association of the higher plasma MDA concentrations with the higher TG concentrations, and, more strongly, with elevated WC and *γ*-GT concentrations. Visceral ectopic fat deposition coexists with hypertriglyceridemia promoting intracellular lipotoxicity, especially in hepatocytes and muscle cells [8]. In hepatocytes, increased fatty acids supply does not essentially result in activation of *β*-oxidation [27]. Hepatocyte accumulation of esterified fatty acids constitutes a stressful stimulus that result in mitochondrial dysfunction with increased ROS production [28], and therefore becoming a crucial situation for liver injury that can be identified by serum *γ*-GT alterations. Elevations in *γ*-GT concentrations is also used to identify chronic alcohol consumption and is related to inflammatory markers [29], blood glucose [30], and metabolic syndrome [31]. In addition, elevations of *γ*-GT concentrations could be an indirect marker of antioxidant response to increased ROS production once *γ*-GT acts on the glutathione metabolism by regulating the oxidized glutathione clearance [32]. This would be possible explication for the strong associations between higher plasma MDA and higher *γ*-GT. However, the precise mechanism to explain this association remains unknown. Different phenotypes of MetS combining hyperadiposity, hyperglycemia and insulin resistance, dyslipidemia, and hypertension results in multifactorial responses such as the high risk for hepatic steatosis, type 2 diabetes, and cardiovascular diseases. Our results showed that hyperadiposity and dyslipidemia are main determinants of higher plasma MDA concentrations, but hyperglycemia and insulin resistance can also contribute to higher MDA concentrations, supporting the hypothesis that MetS-related glucolipotoxicity sets the raising of MDA concentrations in this population. Overall, the lipoperoxidation and MDA formation might be consequence of dysfunctional glycated proteins, AGEs, and glycooxidative stress glycol-oxidative stress (hyperglycemic glycotoxicity) and consequence of lipotoxicity when lipid is forced into organ cells (e.g., liver, skeletal, and heart muscle and pancreas) significantly impairing functions. MetS is a model of metabolism homeostasis breakdown presenting glucolipotoxicity along with abnormalities in blood lipids, glucose, blood pressure, coagulation, and inflammation [33].

Some limitations must be explicit. Antioxidants such as uric acid and plasma HDL-C in the current study did not offer protection against LPO, suggesting that antioxidant protection is important at the time and place of lipoxidation occurrence. So a limitation was the lack of measurement of some fat-soluble vitamins involved in the protection of cell membranes. The practice of physical activity (which is also related to combating inactivity) is known as an important inducer of antioxidant capacity [34]. However, our study subjects were classified as sedentary, the reason that cardiorespiratory fitness values were not used in the adjustment models. Finally, the association found between higher MDA concentrations and the presence of MetS is arithmetical without any causal understanding once this is a cross-sectional approach. Intervention studies focusing on MDA-formation inhibitors must be considered for further investigation in MetS.

## 5. Conclusion

Elevated central adiposity (WC) and *γ*-GT concentrations were the main determinants of the higher plasma MDA concentrations. Hyperglycemia, insulin resistance, hypertriglyceridemia, and higher sugar-intake were also associated with higher plasma MDA concentrations. These markers are directly related to the development of the glucolipotoxic states predisposed by the presence of MetS and seem to be the major determinant of plasma MDA concentration in this pathologic condition. Lifestyle modifications are indicated to these subjects in order to reduce MetS and its comorbidities developments; however, the benefits on higher plasma MDA concentrations are still unknown and it is possible that they will follow modulations on glucolipotoxic states.

## Figures and Tables

**Table 1:** The assessed biomarkers according to groups of plasma malondialdehyde (MDA).

	MDA groups			
	Very low	Low	Increased	Higher
	(<0.593 *μ*mol/L)	(0.593–0.856 *μ*mol/L)	(0.857–1.057 *μ*mol/L)	(>1.058 *μ*mol/L)
	*n* = 37	*n* = 37	*n* = 37	*n* = 37
Anthropometry and body composition				
Body Mass Index (kg/m^2^)	29.6 ± 5.9	30.2 ± 5.6	30.9 ± 6.4	30.8 ± 4.6
Muscle Mass Index (kg/m^2^)	8.1 ± 1.4	8.5 ± 1.5	8.5 ± 1.7	8.4 ± 1.4
Waist circumference (cm)	95.6 ± 13.8	94.0 ± 15.3	96.8 ± 14.6	102.5 ± 13.2∗
Body fat (%)	32.1 (27.2–44.7)	32.3 (29.0–42.9)	32.3 (30.0–45.0)	37.0 (30.6–45.0)
Blood pressures and fitness				
Systolic BP (mmHg)	129 ± 21	127 ± 18	126 ± 14	126 ± 17
Diastolic BP (mmHg)	79 ± 10	79 ± 10	78 ± 11	80 ± 10
VO_2max_ (mL/kg/min)	30.3 ± 7.8	29.1 ± 6.1	29.0 ± 5.8	26.6 ± 5.2
Dietary intake and quality				
Variety (items)	11.8 ± 3.8	13.0 ± 4.2	13.9 ± 3.7	13.3 ± 3.8
Energy intake (kcal)	1197 (892–1801)	1113 (974–1654)	1190 (982–1715)	1575 (1184–1955)∗
Carbohydrates (%)	52.5 ± 10.4	51.5 ± 9.0	54.2 ± 9.3	55.0 ± 11.2
Sugar (servings)	0.5 (0.0–1.8)	0.5 (0.0–2.0)	0.7 (0.0–2.0)	1.0 (0.2–2.5)
Proteins (%)	17.9 ± 6.5	16.4 ± 5.6	17.4 ± 5.0	18.7 ± 5.1
Total fat (%)	29.6 ± 9.1	30.7 ± 14.5	28.3 ± 9.1	30.0 ± 9.5
Saturated fat (%)	8.3 ± 3.4	8.6 ± 5.9	7.7 ± 3.0	7.9 ± 3.8
Monounsaturated fat (%)	9.8 ± 4.1	8.8 ± 4.4	7.9 ± 3.0	10.4 ± 12.9
Polyunsaturated fat (%)	7.9 ± 4.0	7.2 ± 2.9	7.0 ± 2.9	7.6 ± 3.3
Fibers (g)	13.7 (9.0–17.3)	15.0 (10.0–19.2)	13.3 (9.1–20.2)	15.0 (9.4–20.8)
HEI (points)	83.0 ± 13.6	79.6 ± 14.8	78.7 ± 16.0	77.5 ± 12.0
Blood markers				
Glucose (mg/dL)	98.9 ± 42.4	99.5 ± 29.3	97.1 ± 21.2	107.5 ± 31.6∗
HOMA-IR	1.53 (0.91–4.08)	1.47 (1.08–4.01)	1.98 (1.35–3.19)	2.78 (1.33–4.50)
Total cholesterol (mg/dL)	198.9 ± 33.5	200.2 ± 39.3	186.0 ± 42.3	199.3 ± 37.2
HDL-cholesterol (mg/dL)	48.7 ± 11.3	47.6 ± 13.6	48.4 ± 11.5	46.8 ± 11.5
LDL-cholesterol (mg/dL)	122.8 ± 30.9	124.4 ± 36.0	107.7 ± 37.0	118.8 ± 30.9
Triglycerides (mg/dL)	127.0 (108.3–160.8)	133.5 (102.0–180.0)	134.0 (88.0–179.0)	152.0 (106.5–221.5)∗
Uric acid (mg/dL)	4.7 ± 1.6	4.9 ± 1.9	4.9 ± 1.8	5.0 ± 1.4
*γ*-GT (U/L)	23.0 (16.3–38.5)	27.0 (15.8–49.3)	21.0 (17.0–33.0)	33.5 (22.5–46.0)∗
C-reactive protein (mg/L)	2.6 (1.7–7.9)	3.0 (1.7–6.0)	3.3 (1.6–6.3)	3.5 (2.0–6.7)

*Different from other groups (*P* < 0.05).
